# We vote for the person, not the policies: a systematic review on how personality traits influence voting behaviour

**DOI:** 10.1007/s44202-022-00057-z

**Published:** 2023-01-10

**Authors:** Filipe Falcão, Bárbara Sousa, Daniela S. M. Pereira, Renato Andrade, Pedro Moreira, Anna Quialheiro, Carlos Jalali, Patrício Costa

**Affiliations:** 1 Life and Health Sciences Research Institute (ICVS), University of Minho, Campus de Gualtar, 4710-057 Braga, Portugal; 2 ICVS / 3B’s–P.T. Government Associate Laboratory, Braga/Guimarães, Portugal; 3 Espregueira-Mendes Sports Centre—FIFA Medical Centre of Excellence, Clínica Do Dragão, Porto, Portugal; 4 Research Unit On Governance, Competitiveness and Public Policies, Department of Social, Political and Territorial Sciences, University of Aveiro, Aveiro, Portugal; 5 School of Medicine, University of Minho, Braga, Portugal; 6 School of Psychology, University of Minho, Braga, Portugal; 7 Faculty of Psychology and Education Sciences, University of Porto, Porto, Portugal

**Keywords:** Candidates, Personality traits, Turnout, Voting

## Abstract

Western democracies’ voting practices have altered recently, most notably with candidates now taking center stage at the expense of the institutions. These events are the result of a phenomenon called the personalisation of politics. The present review aims to synthesize evidence regarding the impact of voters’ evaluation of candidates’ traits on political outcomes and the effects of voters’ traits on voting. Included studies were identified via electronic databases (up to July 2019). Reviewers extracted data respecting inclusion and exclusion criteria. Methodological quality was assessed independently by two reviewers. Data synthesis was executed through narrative processes. 288 studies were identified, 12 of which were selected for review. Four main outcomes were found: (i) *Personality traits and voting behaviour*; (ii) *Implicit/explicit trait associations and political outcomes*; (iii) *Party identification and personality traits*; and (iv) *Ideology and personality traits*. Data obtained suggests that political outcomes are heavily influenced by voters’ personality traits and how they perceive the personality traits of the candidates. This review advances the theory of personality trait matching and establishes a connection between traits and the personalization of politics.

## Introduction

Elections and political news coverage have changed over the past half-century [1, 2]. Abstention is a political malaise of contemporary democracies and long-term voting variables, such as party identification, are less relevant nowadays [3]. These changes prompted researchers to search for short-term voting predictors. Recent studies on the topic have concentrated on the importance of candidates, who have grown to be prominent to the public and are now more relevant than parties in influencing voting behaviour [4, 5].

This preference for candidates concerns a phenomenon labelled as “personalisation of politics.” Although empirical evidence on the topic is mixed [6–8], a significant part of the literature supports the personalisation thesis, uncovering evidence of its presence in Western democracies [5, 9]. With this in mind, it is pertinent to clarify the consequences of the underlying mechanisms responsible for the exponential growth of this trend in Western politics.

While several studies measuring the effects of personality traits on voting behaviour have emerged in recent years, none provides a systematic analysis on the subject. This review evaluates the literature on the effect of personality traits on voting, considering how existing studies have sought to measure this effect; what results tend to emerge; where the literature yields contradictory results; and what dimensions remain unexplored in the field.

## Background

At the heart of most conceptions of democracy lies the notion of choice. Voters have the option of selecting a party (and the candidates affiliated to the parties) to represent them during elections. Normative conceptions of democracy assume that this choice is based on the different policy stances that parties represent, as individuals vote for those that most closely align with their preferences [10]. However, this conventional trajectory has been shifting in Western democracies [11]. One of the main factors for this change is the weakening of social encapsulation. With levels of education and political interest increasing, the utility of partisan cues has decreased substantially [2]. As a consequence, we are witnessing an increase in the centrality of candidates at the expense of institutions (e.g., parties, cabinets and parliaments) [5, 12].

To explain the relationship between voters’ personality and voting as well as between voters’ evaluations of candidates and the underlying mechanisms that drives voters’ decisions, the existing literature highlights the role of candidates’ and voters’ personality traits [3]. Using a comparable process to the one we employ every day to judge others, voters consider the candidates’ traits as hints regarding their behaviors in decision-making processes and political positions. This phenomenon is frequently referred to as “personalization of politics” and describes a process where the influence of the candidate as an individual is enhanced at the expense of parties [13, 14].

While some authors assert that all traits have similar impact on voting preferences, others contend that some traits are more relevant than others [15]. According to Bittner [16], the traits that appear more often in political surveys are: *leadership, knowledgeable, intelligent, inspiring, honest, trustworthy, arrogant* and *moral*. However, since there are so many variables to consider and the small sample size is limited, it is not practical to examine each trait separately. For this reason, traits are usually grouped within dimensions that serve as the foundation for evaluating candidates [16].

The Stereotype Content Model (SCM) [17] states that we evaluate others through the perception of their intentions (*warmth*) and their capability to make those intentions a reality (*competence*) [18]. Warmth embraces traits such as friendliness, helpfulness, sincerity, trustworthiness and morality; On the other hand, competence reflects traits related to perceived ability, intelligence, skill and efficacy [18]. Both warmth and competence are universal dimensions of social cognition (both at the individual and group level) and govern the judgments we make about others, influencing our emotions and behaviors [19]. The same is true regarding how voters perceive candidates. For this reason, the SCM should, therefore, provide an adequate framework for understanding how voters evaluate candidates based on their traits and how their personality profiles influence voting [18].

The relationships between personality traits and voting decisions has not been extensively studied [20]. As parties take second place, it is relevant to evaluate the impact of candidates’ traits on implicit and explicit measures of voting. Equally, it is relevant to understand how different personality profiles may influence voters. As far as we are aware, no review has been conducted on this subject. By outlining the state of the art, this paper proposes to explore the impact of voters’ evaluation of candidates’ personality traits on political outcomes and the effects of voters’ personality traits on their voting behavior. Furthermore, we aim to obtain an overview of the methodological procedures researchers use to understand this phenomenon and gather homegenous research strategies for collecting data on the topic.

## Methods

This review was conducted according to the Preferred Reporting Items for Systematic Reviews and Meta-Analyses (PRISMA) checklist and flow diagram [21]. Systematic reviews refer to a type of research synthesis that aims to gather data on specific issues, as well as analyse and synthesize the findings of a particular topic to guide practice and future research [22]. The PRISMA checklist and flow diagram are advised reporting methods to prevent errors and advance proper empirical practice [23]. We conducted this review according to a protocol registered in the Open Science Framework’s online repository (available at https://osf.io/dtpha/). The formulation of this protocol was guided by the Preferred Reporting Items for Systematic Review and Meta-Analysis Protocols (PRISMA-P).

## Research questions

This review responds to the following research questions:

1. *How does voters’ evaluation of candidates’ personality traits influence their voting behavior?*
2. *How do voters’ personality traits influence their voting behavior?*

### Data sources and searches

A systematic literature search was conducted from January 2000 to July 2019. The following eletronic databases were searched: SCOPUS-ELSEVIER®, SAGE PUBLISHING® and WEB OF SCIENCE – CORE COLLECTION®. No language restrictions were applied. Search and MESH terms were combined in a search strategy developed for SCOPUS-ELSEVIER. This strategy was adapted for the other databases. Search strategies are presented in appendix 1.

### Study selection/inclusion criteria

Only original studies focused on the topic of interest were included. Experimental and quantitative studies with a longitudinal/transversal evaluation of voting behaviour considering the traits of candidates and voters; studies with community participation; studies with samples composed of individuals of voting age; and studies written in English, Spanish, or Portuguese were considered. Studies that represented grey literature, letters, editorials, commentaries, reviews or conference papers; qualitative studies; and papers that reported incomplete results—of which we did not get a response from the authors in case of attempted clarification—were excluded from analysis.

Research results from database searches were were exported into Rayyan Management Software [24]. Study selection, data records, search results and eligibility criteria were conducted within the software. Duplicates were removed. Article screening was conducted in two stages. First, two authors (BS, FF) independently screened citations on titles and abstracts. Results were compared and disagreements discussed. Agreed citations were retrieved in full text and screened independently by two authors (BS, FF). Full-text citations were considered based on the inclusion and exclusion criteria discussed above. Disagreements were discussed and resolved by consensus; if an agreement was not reached, a third author was consulted for a definitive decision (PC). Retrospective and prospective snowball citation were conducted to achieve literature saturation.

### Data processing and quality assessment

Data from included citations was extracted by two reviewers (FF, BS) using a purpose-built data extraction form (Cf. Appendix 2). Methodological quality of included studies was measured considering the risk of bias (RoB). Two reviewers (FF, BS) evaluated the RoB in each study using the Risk of Bias Assessment tool for Non-randomized Studies (RoBANS) [25]. RoBANS is harmonized with the the Cochrane’s RoB tool and GRADE (Grading of Recommendations Assessment, Development and Evaluation) and is proper for assessing RoB of included studies in systematic reviews [25]. Inter-rater reliability of the RoB evaluation was measured using the Cohen’s kappa coefficient (κ).^1^ Appraisals were matched and disagreements were discussed. When a consensus was not met, the last author (PC) served as tiebreaker. RoB and inter-rater reliability assessment strategies are detailed in appendix 3.

### Data synthesis

Citations included for review were summarised to draw valid conclusions [26]. Due to the high degree of heterogeneity found among the included studies in terms of study design, population, adjustments, measures of association and statistical procedures, statistical meta-analysis was not feasible [27]. This limitation was overcome by using narrative synthesis to conduct the data synthesis process. By adopting a textual approach to the synthesis procedure, a narrative of the findings from the included citations is built, which enables the review to concentrate on a wide range of issues beyond just the efficacy of a particular intervention [28].

### Results

**Fig. 1:**
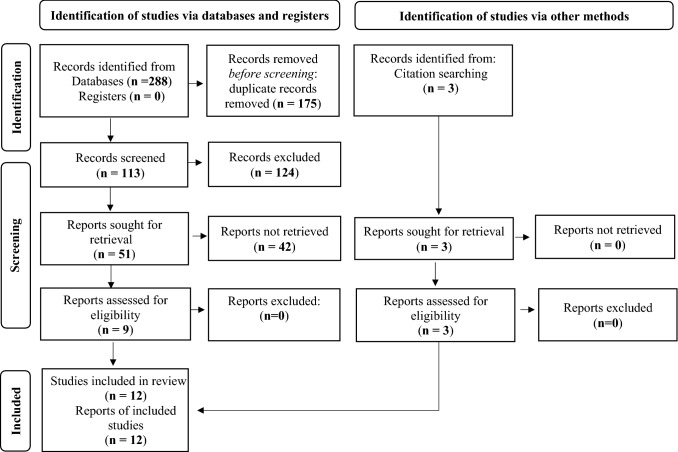
PRISMA flow diagram of included studies

**Fig. 2:**
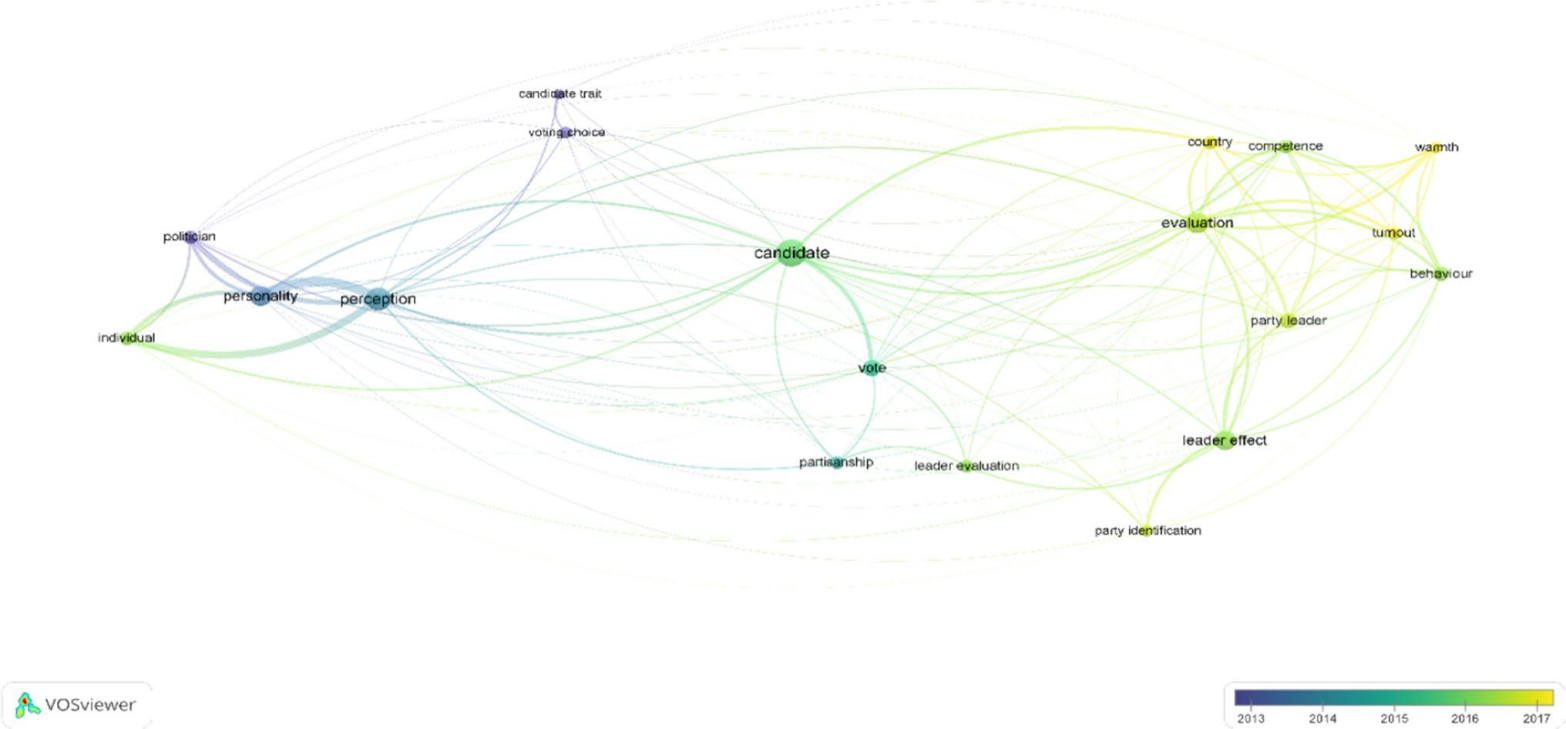
Overlay map-based visualization on text data retrieded from title and abstract fields during full text screening. The color bar reflects how the publication year of the articles is mapped to colors. The nodes reflect the words that were most frequently used in the title and abstract fields of the literature that was screened in full

Searches retrieved 288 studies, 175 of which were unique. Following title/abstract screening, 121 citations were removed, leaving 51 full-text articles for further consideration. Of those, 41 were not eligible since they did not cover the topic under study. The remaining nine citations met the criteria to be eligible for review. Retrospective and snowball citation tracking added 3 articles for review. In total, 12 studies were included. A PRISMA diagram depicting this process is presented in Fig. 1. An overlay map-based visualization based on the text data retrieved from the title and abstract fields during full-text screening is presented in Fig. 2.

#### Characteristics of included studies

A detailed description of the included studies is provided in Table 1. Eligible studies were published from 2006 and 2019. Seven studies were conducted in the USA [29–35], two in Portugal [3, 36], one in England [37] and one in Belgium [38]. One study conducted a multinational experiment in Spain and Italy [39]. All included studies presented a quantitative nature. Most studies implemented a cross-sectional design except two studies, which reported data from a longitudinal approach [34, 37]. The median sample size was 3498 people (range 109 to 77021 participants). The median age of participants was 47.4 years (range 34–52.3).

**Table 1:** Description of included studies

First author, year	Country	Purpose	Study design	Outcome	N	Method	Findings
Aidt and Rauh [37]	England	Authors proposed a framework for distinguishing the effects of transitory events and individual characteristics on party identification	Longitudinal	Party identification and personality traits	77,021	Authors related the stable component of partisanship to personality traits. They estimated a linear probability model with individual, region and time fixed effects to partisanship. Estimates of the stable component of party preference were the outcome variable that the authors related to the measures of the Big Five personality traits	Personality traits exerted effects on the stable part of party preference. Authors observed differences between the traits of the supporters of the three major parties in England (Labour, the Conservatives, and the Liberal Democrats): Conservatives presented low levels of Agreeableness, low Openness, high Extraversion, and low Neuroticism Supporters of the Labour Party presented high Agreeableness, high Openness, high Neuroticism, and low Conscientiousness Liberal Democrats presented insignificant Agreeableness and low Extraversion
Avery, Lester and Yang [29]	USA	Examine the influence of personality traits on vote share	Cross-sectional	Personality traits and voting behaviour	619,397	Authors regressed Obama’s vote share in the 2008 and 2012 American Elections on the big five personality traits and other political variables in different models	The first models predicted Obama’s vote share for 2008 and 2012 without including political variables and white racial prejudice as controls: agreeableness and openness had positive and significant effects on Obama’s vote share Remaining models predicted Obama’s vote share by controlling for citizen liberalism, policy mood, Democratic Party identification, and white racial prejudice. Effects of agreeableness and openness were not statistically significant Direct effects of personality traits on Obama’s vote share were found: Extraversion had positive effects on citizen liberalism in 2008, but not in 2012; Agreeableness had no significant effect on the political variables or white racial prejudice. Conscientiousness had negative effects on citizen liberalism and policy mood, and positive effects on white racial prejudice; Neuroticism had positive effects on white racial prejudice but no effect on citizen liberalism or policy mood Indirect effects of personality traits on Obama’s vote share were found: Conscientiousness could lead to lower support for Obama through negative effects on citizen liberalism and policy mood and positive effects on white racial prejudice; Openness could lead to greater support for Obama through positive effects on citizen liberalism, policy mood, and Democratic Party identification, and negative effects on white racial prejudice
Barker, Lawrence and Tavits [31]	USA	Explores whether Democratic voters emphasize different traits when evaluating party nominees than Republican voters	Cross-sectional	Party identification and personality traits	Study I: 767 Study II: 2214	Authors developed models which were estimated using binary logistic regression analysis. Authors controlled for other possible influences on vote choice such as media/campaign exposure, age, education, race, sex, and personal income	Democratic primary voters tend to rely on perceived candidate compassion. Republicans are more likely to rely on perceived personal virtue
Costa and Ferreira da Silva [36]	Portugal	Examine how the evaluation of candidates’ traits influences voting behaviour	Cross-sectional	Implicit trait associations and political outcomes	7134	Multiple sequential binary logistic regression models were performed to analyse the predictive power of competence and warmth on voting behaviour	Block 1: Education was the only significant socio-demographic variable Block 2: All the country dummies were statistically significant Block 3: Party identification remained the feature of most importance Block 4: No significant effects of gender, age and education were found. Authors found significant effects for Portugal, UK, and for Hungary, but in the opposite direction. Spain, Ireland, and Italy did not differ significantly from Germany. Ideology was the main predictor of voting behaviour. Warmth and competence evaluations of left-wing party leaders were statistically relevant in distinguishing left/right vote. These results were not true for right-wing party leaders, where only warmth had a significant effect
Ferreira da Silva and Costa [3]	Italy	Examine how the evaluation of party leaders’ traits influences voter turnout in General elections	Cross-sectional	Implicit trait associations and political outcomes	8.188	Multiple binary logistic regression models were performed to analyse the predictive power of competence and warmth on turnout, controlling for sociodemographic, political ideology variables and voters past political behaviour	Results reveal the relevance of warmth personality traits of leaders in voter turnout decisions. Effect of competence were lost once controls were introduced. Interaction effects were demonstrated between warmth evaluations and identifying with a right-wing party as well as past political behaviour with both warmth and competence
Gerber et al. [30]	USA	Examine the associations between personality traits and the strength and direction of partisan identification	Cross-sectional	Party identification and personality traits	12,000	Authors developed models where the dependent variable was Party Identification. Authors assessed the relationships between dispositional traits and directional party identification	Associations between the Big Five and the direction of partisan identification were mediated by ideology Personality traits shape both the decision to affiliate with a major political party and the strength of that affiliation Individuals higher on Conscientiousness and Emotional Stability report being more Republican (conservative), whereas respondents higher on Openness report being more Democratic (liberal) The affective, social benefit of partisan affiliation would be greater for individuals higher on Extraversion and Agreeableness. Partisan attachments provide are particularly appealing to individuals low on Openness and Emotional Stability and high on Conscientiousness
Holian et al. [33]	USA	Examine the effect of perceptions of candidates’ personality traits on vote choice	Cross-sectional	Implicit trait associations and political outcomes	3485	Authors presented data on the perceptions of candidate traits from the 2012 American elections. Authors employed logistic regressions models of the vote	Model 1: Both variables were statistically significant Model 2: evaluations of Obama’s as president improved the predictive ability of the regression and diminished the coefficients for party identification and ideology Model 3: issue indices and the index of attitudes toward blacks were significant Model 4: overall candidate trait index improved model fit and increased the pseudo-R2. Trait perceptions weakened the influence of the evaluations of presidential performance Leadership and empathy were the most important traits in the elections. Independents were the party most affected by trait perceptions. Democrats and Republicans were affected as well, but they had different concerns: republicans cared more about having a president who was a strong leader and was trustworthy; Democrats wanted someone who understood and cared about the average American
Ksiazkiewicz, Vitriol and Farhart [34]	USA	Examine implicit candidate trait associations effects on political outcomes	Longitudinal	Implicit trait associations and political outcomes	714	Authors used a time-series cross-sectional model for clustered data analysis to examine whether implicit trait associations of warmth and competence accounted for unique variance in political outcomes	Authors found evidence of cross-sectional, between-participant effects for implicit competence associations on evaluations of candidates and the economy. Implicit warmth had a significant cross-sectional effect on candidate evaluations, but it did not have a significant effect on evaluations of the economy When controlling for implicit competence, the effect of implicit warmth on candidate evaluations was not significant. Implicit candidate-warmth associations play an inferior role in candidate evaluations than implicit competence and no role in evaluations of the economy Implicit trait results parallel the explicit level
Roets and van Hiel [38]	Belgium	Explore the relationship between voters’ ideology and their image of politicians’ ideal personality	Cross-sectional	Ideology and personality traits	109	Authors conducted ANCOVAs with the five-factor model personality dimension scales as a within-subject factor. Political ideology was included as a continuous, between-subjects factor. Finally, a SEM was performed to test whether the predictive value of political orientation on the political leadership traits was mediated through openness and agreeableness	Personality and ideology yielded significant effects on political orientation. A significant interaction effect between personality and ideology was obtained Conscientiousness was more valued than any other trait. Neuroticism was the least valued trait in the ideal politician image The degree to which voters consider some traits important depends on their ideology: conscientiousness is most valued for both left-wing voters and right-wing voters. Neuroticism is the least valued trait
Vecchione, Castro and Caprara [39]	Italy & Spain	Examine the relationship between voting choice and similarity in traits between voters and candidates	Cross-sectional	Party identification and personality traits	1051	Authors conducted t-tests for independent samples to measure differences in self-reported personality traits among voters of the two major parties in Italy and Spain. To measure similarity, authors estimated the generalized Euclidean distance measure between the personality ratings of the participants and the politician evaluated across the entire set of adjectives. Next, authors conducted t-tests for dependent samples to determine whether voting for a party was associated with a greater similarity to the personality of the candidate	Spain: *Voters’ personality traits:* markers of Openness and one marker of Emotional stability were significantly higher among PSOE voters compared to PP voters *Perceived politicians’ traits:* Seventeen adjectives revealed significant differences between candidates. All markers of Energy/Extraversion, four markers of Openness, three markers of Emotional stability, and two markers of Agreeableness were perceived as higher in Zapatero *Similarity between voters and candidates:* voters were most likely to see themselves as similar to the political leader of their preferred party Italy: *Voters’ personality traits:* PDL voters scored higher than PD voters on three markers of Energy/ Extraversion, and three markers of Conscientiousness *Perceived politicians’ traits:* Berlusconi and Veltroni revealed significant differences in twenty-one adjectives. All markers of Energy/ Extraversion, three markers of Emotional stability, one marker of Openness, and one marker of Conscientiousness were perceived as higher in Berlusconi. All markers of Agreeableness, three markers of Conscientiousness, two markers of Emotional stability, and one marker of Openness were perceived as higher in Veltroni *Similarity between voters and candidates: v*oters were most likely to see themselves as similar to the political leader of their preferred party. Similarity was higher for markers of Energy/Extraversion and Agreeableness
Vitriol, Ksiazkiewicz and Farhart [35]	USA	Examine whether implicit trait associations shape explicit political judgment, preferences, and behaviours	Longitudinal	Implicit trait associations and political outcomes	247	Authors used weighted regression models to estimate incremental effects of implicit warmth and implicit competence on continuous dependent variables	Implicit competence-associations were a robust predictor of political evaluations above and beyond the impact of explicit competence, demographic variables, and partisan identifications. Implicit warmth association had small predictive value above and beyond the role of explicit associations, demographic variables, or partisan identification Implicit competence- associations were robust predictors of vote preferences, above and beyond explicit competence measures, demographics, and partisan identification. Implicit effects are consequential for electoral outcomes. Implicit warmth associations are less consequential for vote preference when explicit warmth assessments are included in the model
Wang [32]	USA	Examine the mediation effect of the Big Five personality traits on vote choice	Cross-sectional	Personality traits and voting behaviour	3349	Authors estimated direct and mediation effects of personality traits on vote choice controlling for demographic factors	Effects of traits on vote were mediated by party identification, feelings towards the candidate, policy preference and executive approval Extraversion, conscientiousness, and emotional stability had negative mediation effects on voting decisions. Openness to experience exerted positive mediation effects through party identification, feelings toward candidates, policy preference and executive approval Extraverted and conscientious voters tend to possess Republican identification. Voters open to experience tend to have Democratic identification

#### Quality appraisal

A summary of a RoB table and a RoB ratings for each citation is included in Table 2. Four studies were evaluated with a high RoB. Reviewers presented concerns in one study and classified seven studies with low RoB. Main sources of bias found were the following: majority of studies presented a cross-sectional design, which prevented causal relationships between the variables in investigation; no study reported participation rates; almost all studies failed to justify the sample size used or provide estimates of effects of variances; four studies [3, 31, 32, 36] used the same dataset; two studies [38, 39] lacked representativeness since they used convenience sampling; most studies [3, 31, 33–36] did not use specific self-report measures to assess candidates’ traits; only one study [36] presented detailed information on the measures employed.

**Table 2:**
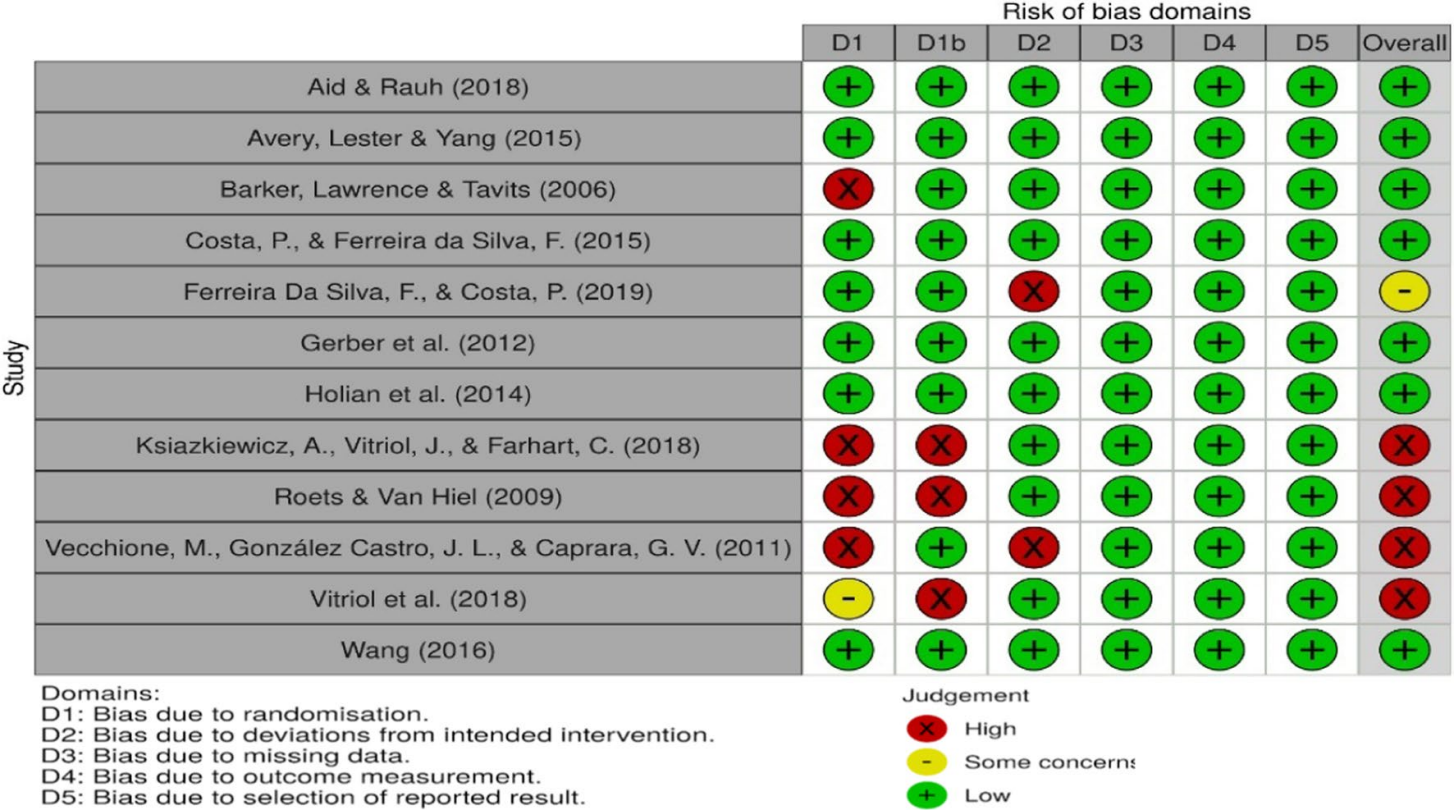
RoB table and RoB ratings for the included studies

#### Outcomes

Different outcomes were reported: (a) personality traits and voting behaviour [29, 32]; (b) Implicit/explicit trait associations and political outcomes [3, 33, 36]; (c) Party identification and personality traits [30, 31, 37, 39]; and (d) ideology and personality traits [38].

#### Outcome measures

Effects of personality traits on voting were measured using self-report measures, the Implicit Association Test (IAT) [40], the Ten-Item Personality Inventory (TIPI) [41] and the Big-Five Inventory (BFI) [42]. Effects of implicit/explicit trait associations on political outcomes were measured using self-report measures and the IAT. Relationships between personality traits and party identification were measured using self-report measures and the TIPI. Relationships between ideology and personality traits were measured using self-report measures and an adapted version of the NEO Five-Factor Inventory [43].

#### Findings

##### (a) Voters personality traits and voting behaviour

Two studies [29, 32] analysed the effects of voters’ personality traits on voting. Both used the Big Five framework [43] to measure the influence of personality traits. The Big Five encompasses a broad range of attitudes and behaviors across several domains that can be described by five major dimensions of personality—*extraversion, agreeableness, conscientiousness, emotional stability* and *openness to experience* [43]. Although the Big Five is not associated with political attitudes and behaviors, it includes broad dispositions that shape how individuals react to events. For this reason, scholars use this framework to examine how the traits of voters and politicians affect political outcomes [44].

Avery et al. [29] examined voters’ personality traits on presidential vote share in the 2008 and 2012 American Elections. Results revealed mediating paths via which traits were linked to Barack Obama’s vote share: conscientiousness had a negative effect on citizen liberalism and policy mood and a positive effect on white racial prejudice; neuroticism had a positive effect on white racial prejudice, but no effect on citizen liberalism or policy mood; and agreeableness had no significant effect on the political variables or in white racial prejudice. When measuring for indirect effects, the authors found that conscientiousness could lead to lower support for Obama due to its negative effect on citizen liberalism and policy mood and its positive effect on white racial prejudice. In the same vein, openness could lead to greater support for Obama through its positive effect on citizen liberalism, policy mood, and Democratic Party identification, and its negative effect on white racial prejudice.

Wang [32] examined mediation effects of voters’ personality traits on voting. The study reports that the impact of personality traits on vote choice is mediated by factors such as party identification, feelings towards candidates, policy preference and executive approval. The author contends that extraversion and conscientiousness have negative mediation effects on vote choice, while openness to experience positively affects voting through party identification, feelings toward the candidates, policy preference, and executive approval. Extraverted and conscientious voters tend to prefer Republican candidates while having negative feelings toward the Democratic candidates. In the same sense, voters who are open to experience possess Democratic identification and positive feelings towards Democratic candidates.

##### (b) Implicit/explicit trait associations and political outcomes

Five studies [3, 33–36] examined how implicit evaluations of warmth and competence of political candidates may influence political outcomes. All authors were unanimous in setting leaders in mainstream political outcomes such as the vote, reinforcing the importance of candidate trait evaluations. The only point of contention between the authors was related to the importance given to both dimensions, with part of the authors highlighting a greater role for competence, while others emphasized the importance of warmth.

Ksiazkiewicz and colleagues [34] revealed evidence of cross-sectional between-participant effects for implicit competence associations on vote choice and evaluations of candidates. The authors found that implicit warmth had significant cross-sectional effects on candidate evaluations. However, when controlling for implicit competence, the effects of implicit warmth on candidate evaluations were not significant. The authors report that implicit warmth-associations play a smaller role than implicit competence in different political outcomes, reinforcing the importance of competence-associations.

Vitriol and colleagues [35] revealed that implicit evaluations have an impact on electoral outcomes. According to the authors, implicit competence-associations represent robust predictors of explicit evaluations of candidates, demographic variables and partisan identifications. Congruently with Ksiazkiewicz and colleagues [34], the authors claim that implicit warmth associations reveal little predictive value of explicit associations, demographic variables or partisan identification. Although both studies recognize that implicit warmth-associations are related to voting preferences, they are less consequential, especially when explicit warmth evaluations are considered.

In contrast, Costa and Ferreira da Silva [36] reinforced the personalisation theory by revealing the importance of candidates’ warmth. The authors examined how the evaluation of candidates’ traits influenced voting and found that warmth and competence evaluations of left-wing party leaders are statistically relevant in distinguishing a left or right vote. The same is not true for right-wing party leaders, where only warmth is significant. Although ideology remained the main predictor of voting, the authors report that the candidates’ traits are significant predictors of voting as well, even after controlling for socio-demographic variables and ideology.

Congruently, Ferreira da Silva and Costa [3] examined how the evaluation of candidates’ traits influences turnout in general elections. The study emphasizes the importance of candidates’ warmth traits in turnout decisions and found that effects of competence on turnout are lost once controls variables are introduced in the model. The authors went further, finding interaction effects between warmth evaluations and the identification with right-wing parties and past political behaviour with both warmth and competence evaluations.

Finally, Holian et al. [33] reported that trait perceptions weakened the influence of evaluations of presidential performance, with leadership and empathy being the most important traits in the 2012 American elections. Independents were the partisan group most affected by trait perceptions. Democrats and Republicans were affected as well. However, they revealed different concerns: republicans cared more about a president who was a strong leader and trustworthy; Democrats wanted a president who understood the average American.

These studies demonstrate the existence and relevance of implicit candidate-trait associations on political outcomes. They represent evidence of incremental effects of implicit associations above the role of explicit counterparts and conventional controls. These findings point to the importance of personality traits in political outcomes and the need to understand how the connection between trait judgements and political behaviour requires the deliberation of both implicit and explicit processes of candidate evaluations.

##### (c) Party identification and personality traits

Four studies measured the effects of traits on party identification [30, 31, 37, 39]. All studies claim that traits exert effects on party identification. All authors, except for Barker, used the Big-five framework to measure personality traits and observed systematic variations in how those traits affected party identification in different countries.

Aidt and Rauh [37] pointed out differences between the traits of the core supporters of the three major political parties in England (Labour, the Conservatives and the Liberal Democrats). According to the authors, supporters of the Conservative party present low levels of agreeableness, openness and neuroticism and high levels of extraversion and conscientiousness. On the other hand, supporters of the Labour party present high levels of agreeableness and openness. However, they present higher levels of neuroticism and low levels of conscientiousness. Supporters of the Liberal Democrats presented similar traits to the Labour supporters with two exceptions: insignificant agreeableness and low extraversion. Individuals without strong identification with any party were similar to those of the supporters of the Conservative Party.

Barker et al. [31] and Gerber et al. [30] focused on the effects of traits on party identification in the USA. According to Barker and colleagues, Democrats emphasize the perceived candidate compassion, whereas Republicans tend to rely more on perceived personal virtue. Gerber’s study goes further, claiming that traits influence how individuals affiliate with parties and the strength of that affiliation. According to the authors, extraversion, agreeableness, and openness can predict the strength of partisan identification and the decision to affiliate with a party. This holds true even after controlling for ideology and a variety of issue positions since associations between the Big Five and the direction of partisan identification are mediated by ideology. The affective, social benefit of partisan affiliation was greater for individuals with higher scores on extraversion and agreeableness. In contrast, partisan attachments were more appealing to individuals low on openness and emotional stability and high on conscientiousness. Furthermore, individuals with higher levels of conscientiousness are closer to the Republican party, whereas respondents with high levels of openness are closer to the Democratic.

Vecchione and colleagues [39] examined the relationship between voting and similarity in traits between voters and candidates from Spain and Italy. The authors found that voters were most likely to see themselves as similar to the candidate of their preferred party in both countries. In two different studies, the authors found that voters from the Spanish Socialist Workers' Party (PSOE; Social democratic) presented higher levels of openness than voters from the People’s Party (PP; Conservative). Congruently, the candidates from the PSOE presented higher levels of extraversion, openness, and agreeableness than the candidates from the PP. The case differs in Italy, with supporters from the People of Freedom (PdL; Conservative) presenting higher levels of extraversion and conscientiousness than the Democratic Party (PD; Democratic) supporters. In the same sense, candidates from the PdL presented higher levels of extraversion, openness and conscientiousness than the candidates from the PD.

##### (d) Ideology and personality traits

Roets and Van Hiel [38] explored the relationship between voters’ ideology and their image of a politicians’ ideal personality in terms of the Big Five and different political leadership dimensions from the Gough Adjective Checklist [45]. The ideal candidate's personality proved to be influenced by both ‘ideology-free’ preferences and ‘ideology-driven’ preferences. The authors found that the interaction between personality and ideology had a significant impact on political orientation. This interaction revealed that the degree to which voters consider traits important depends on their ideology. Conscientiousness and extraversion were the most valued traits for left and right-wing voters. At the same time, openness, agreeableness, conservatism, friendliness, pacifism and achievement drive depended largely on voters’ political orientation: right-wing ideology was positively associated with a higher perceived value of Machiavellianism, achievement drive, and conservatism; while left-wing ideology was positively associated with a higher perceived value of friendliness and pacifism.

#### Findings’ interpretation

Twelve citations were synthesized. Most studies investigated the effect of implicit/explicit trait associations on political outcomes. All studies provide evidence that, in aggregate, shines light on whether traits, either of voters or candidates, can influence voting. Data obtained provides support that voters' assessment of candidates' traits influences different political outcomes and that voters' traits have repercussions on their voting outcomes. Key findings of this paper are summarized on Table 3.

**Table 3:** Summary of findings

Trait/dimension of traits	Main findings
Warmth	•Plays significant cross-sectional effects on candidate evaluations when not controlling for implicit competence [34] •Little predictive value of explicit associations [35] •Warmth evaluations of left-wing party leaders are relevant in distinguishing a left or right vote [36] •Determinant in voter turnout decisions [3]
Competence	•Significant cross-sectional effect on candidate evaluations [34]; •Implicit competence associations are predictors of explicit evaluations of candidates [35] •Competence evaluations of right-wing party leaders are relevant in distinguishing a left or right vote [36]
Openness to experience	•Could lead to greater support for a democratic candidate through its positive effect on citizen liberalism, policy mood, Democratic Party identification, and its negative effects on white racial prejudice [29]; •Positively affects voting behaviour through party identification, feelings toward candidates, policy preference, and executive approval [32] •Low in Conservative party supporters and individuals without strong party identification [37] •High in Social democratic, Labour and Liberal democratic party supporters + [37, 39] •Predicts the strength of partisan identification [30]
Conscientiousness	•May indirectly lead to lower support for a democratic candidate due to its negative effects on citizen liberalism and policy mood and positive effects on white racial prejudice [29]; •Negative mediation effects on vote choice [32] •High in Conservative party supporters and individuals without strong party identification [37, 39] •Low in Labour and liberal democratic party supporters [37] •Valued by left and right-wing voters [38]
Extraversion	•Negative mediation effects on vote choice [32] •High in Conservative party supporters and individuals without strong party identification [37, 39] •Low in Liberal democratic party supporters [37] •Predicts the strength of partisan identification [30] •Valued by left and right-wing voters [38]
Agreeableness	•Low in Conservatives [37] •High in Labour party supporters [37] •Predicts the strength of partisan identification [30]
Neuroticism	•Positive effects on white racial prejudice [29] •Labour party supporters presents high levels of neuroticism [37]

## Discussion

Due to societal modernization, changing media logic, growing de-ideologization, and the resulting partisan dealignment trend, short-term factors are becoming structural predictors of voters' choices [46, 47]. By living in a context of progressive party dealignment and pervasive mediatization of politics, voters tend to cast their ballots based on the evaluations they make of candidates, which are no longer perceived as mere party figureheads [2, 46, 48].

The study of personality traits and politics has received renewed interest by the scientific community in the last decades [49]. Despite the work done in the field, insufficient research has been done on how voters decide between candidates. Studying the complexity of this process is relevant because it could shed light on how candidates affect voter attitudes [50]. In an attempt to explain this relationship, scientists have focused on the role that traits of both voters and candidates play in voting decisions [3]. However, existent research has failed to assess whether traits play a role in electoral results or not [51]. The objective of this review was to summarize the literature focused on the impact of voters’ evaluation of candidates’ traits on political outcomes and the effects of voters’ traits on voting decisions.

### Voters’ personality traits and voting behaviour

Voters’ personality traits represent significant predictors of voting decisions [32]. Most of the reviewed literature used the Big Five framework to study the influences of voters’ traits on voting behaviour, revealing effects and the predominance of determined trait patterns in voters, considering the party they support [52]. This is consistent with the vast majority of the literature on politics and personality, which frequently use the same framework [52–55]. According to the studies included for review, high levels of extraversion and conscientousness may have negative mediation effects on vote choice. However, extraversion seems to predict the strength of partisan identification and the decisions to affiliate with parties [56]. On the other hand, openness to experience reveals positive mediation effects (mainly through party identification, candidates thermometers, policy preferences and executive approval), predicting the strength of partisan identification and party affiliation. Neuroticism and agreeableness had no significant effects on vote choice. The data obtained also revealed differences in the personality patterns between supporters of different parties and ideologies. Voters are most likely to perceive themselves as similar to the candidate of the party that they support: conservative party supporters demarcate themselves by having elevated levels of extraversion and conscientiousness; while democratic supporters present prominent levels of agreeableness and openness. Extraversion is associated to collective and individual political activities and is the most relevant trait for both left and right-wing voters in terms of ideology [56].

### Evaluation of candidates' personality traits and political outcomes

The data obtained in this review provides evidence that sustains the personalization of politics thesis in Western politics [11, 57]. Candidates are now key players in politics and the evaluation of their personality traits is a major factor in political outcomes [11]. Voters use candidate’s personality traits as cues about how they will act in political situations and when making decisions [4]. Retrieved literature reports that candidate trait evaluations influence political outcomes, enhancing the candidate’s power over political outcomes. This assessment is primarily based on dimensions of traits [16]. Majority of retrieved citations based their work on the SCM framework, analysing the consequences of explicit/implicit evaluations of warmth and competence on political outcomes. Both warmth and competence represented robust short-term predictors of evaluations of candidates, turnout decisions, demographic variables and partisan identifications [3, 36]. However, there seems to be uncertainty as to which dimension has a more predominant role in the electoral outcomes above-mentioned, with one section of the literature focusing on the importance of warmth, the other on the importance of competence.

### Implications for the political sphere

Electoral decisions based on traits can have consequences for democratic functioning [54]. One should consider that candidate evaluations, if dependent only on the personal characteristics of the parties’ frontrunners, may potentiate volatile turnout rates and endanger the structuration of party competition, as electoral participation will be more dependent on the selection of proper political personnel [58]. This may cause parties to nominate candidates who are perceived as appealing to the electorate, personalizing campaigns to give them visibility and capitalize on their mobilizing potential [48]. Additionally, one must also consider the personality connections between candidates and voters. It is known that individual candidates face electoral incentives to personalise their campaigns and communications with the electorate [12, 59]. Along with the media's assistance, political candidates may run personalised election campaigns [12]. As a consequence, campaign rhetoric and promises may be favoured at the expense of the parties [60]. Candidates may place their personality front and center, using their traits to influence voters and downplaying criteria related to ideology, policy and political experience [61]. Scholars should be concerned and try to identify the type of voters and the type of leaders that may exhibit particular dispositions (e.g., authoritarian perspectives) that may bring disarray to democratic governance [61, 62].

### Strengths and limitations

This review's systematic nature can be considered a strength of this paper. Efforts were taken to ensure transparency and improve trustworthiness during the review process. Using a robust search strategy, followed by back and forward citation tracking, together with proper quality assessment procedures, prevented potential publication bias and enabled the synthesis of relevant studies on the topic of interest.

Despite these strengths, there are also limitations to note. First, one should report the absence of a meta-analysis to combine the results of the included studies for review. Our intention was to include a meta-analytic procedure to synthesise data. However, this type of analysis was not possible due to the wide heterogeneity between the studies considered for review. Secondly, the number of studies considered was small. Third, we reviewed studies with overlapping databases. Fourth, the plurality of instruments used in studies to measure personality traits may also be a limitation, in that there is no single framework to interpret the effects under study. Although much of the studies included for review use the Big Five model, some studies measured personality traits without the use of specific instruments. Fifth, one should consider the time gap between the reviewed literature and the time of publication of this paper. This difference has to do with possible biases, both in the evaluation of the candidates and in the personality picture of the voters themselves. Due to the pandemic context in which we are inserted, evolutions may have occurred in the personality traits exhibited by the candidates and the voters that were not present in the pre-pandemic. Sixth and finally, we did not exclude studies with poor overall assessment due to the small number of studies used for the review.

Despite these limitations, to the best of our knowledge, this is the first systematic review to look at the connection between voters’ personality and voting behaviour and between voters’ evaluations of candidates and the underlying processes that drives voters’ decisions. The analysis in this paper has expanded the existent literature on the role of traits in voting decisions. A comprehensive overview on how traits act as drivers of voting behaviour and which traits are most relevant was provided.

### Directions for future research

Results obtained open new avenues for further research. Longitudinal studies that track major macro-political events and the role that personalization may play in voting behavior would be relevant to investigate the preponderance of this phenomenon. Future studies should focus on whether these findings hold in countries where personalization does not manifest itself as much as in Western policies [48]. Studies that address personality development in candidates from the time they begin campaigning until the end of their terms in office would be important to understand possible attitudinal changes and their effects on the electorate. Moreover, future studies should deepen the psychological mechanisms underlying personality and its role on voting outcomes. Another pertinent point for future research concerns the political changes caused by the COVID-19 pandemic. The pandemic is a dramatic global public health challenge that originated political crisis in many countries due to the economic consequences of unemployment and public sector spending [63]. However, despite this significant impact, insufficient attention has been given to the challenge posed by the pandemic to the legislatures [64]. Future studies should focus on the impact of the pandemic on the political context and the candidates themselves. With the crisis policies imposed in an alarming situation like the one we are living, voters' preference for candidates with certain types of traits may well be different from what it would be in a pre-pandemic context. In the same sense, whether out of obligation or the pressure to respond to the crisis, the candidates themselves may have changed personality traits or chosen a different strategy when it comes to the traits they expose to the electorate during these challenging times.

## Conclusion

Democracies have become more individualized and party leaders are now the decisive actors in the political system. Data obtained presents consequences for the personalization of politics on democratic functioning. Voters' evaluation of candidates’ traits represents significant determinants of the vote. In the same vein, voters' traits are a major factor in voting outcomes. Through this review, we looked at the impact that personality traits of both voters and candidates can have on voting and how the personalization framework may help understand this increasingly present trend in Western politics. A thorough overview of the personality traits that influence voting behavior was provided. We add to the theory of personality trait matching and build a link between traits and the personalization of politics phenomena.

## Data Availability

Data sharing not applicable to this article as no datasets were generated or analysed during the current study.

## Appendix 1

A two-step search strategy was used to identify relevant studies. First, a literature search was conducted within the SCOPUS-ELSEVIER®, SAGE PUBLISHING® and WEB OF SCIENCE—CORE COLLECTION® electronic databases between January 2000 and July 2019. The search used terms combined into the following query: (candidate*) OR (leader*)) AND TITLE-ABS-KEY ((traits) OR (traits AND of AND personality) OR (leader's AND personality AND traits) OR (personalization AND of AND politics) OR (leader AND effect*)) AND TITLE-ABS-KEY ((vot*) OR (turnout) OR (voting AND behavior)) AND TITLE-ABS-KEY ((political AND ideology) OR (party AND choice))) AND PUBYEAR > 1999. Second, studies retrieved in the first step were supplemented by a back and forward citation tracking and consultation with experts regarding the topic of interest.

## Appendix 2

For each study, we identified the author(s) name(s), year of publication, study design, and measure used to assess candidates’ personality traits. Data regarding sample size (*N*), sociodemographic characteristics (i.e., mean age and gender of participants) was collected and study procedures were summarized. Personality dimensions (voters and/or candidate) and party dimensions (i.e., party choice and/or party identification) were identified. Results regarding the effect of candidates’ personality traits on voting behaviour, considering the ideology of the chosen candidate, were extracted through descriptive statistics of voting behaviour and party identification variables (i.e., left, right), correlation among measures, regressions analysis and mediation analysis.

## RoB assessment strategy

RoB of each study was assessed in terms of six domains of the RoBANS tool: (D1) selection of participants; (D1b) confounding variables; (D2) measurement of intervention (exposure); (D3) blinding of outcome assessment; (D4) incomplete outcome data; and (D5) selective outcome reporting. RoBANS includes criteria for judging the RoB for each domain with one of three ratings (‘Low’; ‘High’; or ‘Some concern—unclear’). If at least two domains were evaluated with a ‘High’ or ‘Unclear’ RoB, the study was considered to have a high RoB.

## Inter-rater reliability assessment strategy

Cohen’s kappa coefficient (κ) values ranged from − 1 to + 1, with values below 0 representing no agreement; 0.01–0.20 representing none to slight agreement; 0.21–0.40 representing fair agreement; 0.41–0.60 representing moderate agreement; 0.61–0.80 representing substantial agreement; 0.81–0.99 representing almost perfect agreement, and 1 representing perfect agreement [65].

## Footnotes

1 Inter-rater agreement analysis was conducted using R (Version 4.0.3), R studio Desktop (Version 1.4.1103) and R package ‘Irr’ (Gamer et al., 2019).
